# Utero-ovarian transposition before pelvic radiation in a patient with rectal cancer: a case report and systemic literature review

**DOI:** 10.3389/fsurg.2024.1336047

**Published:** 2024-02-26

**Authors:** Daniela Huber, Colin Simonson, Ian Fournier, Irma Dischl-Antonioni, Francisco Javier Pena Rios, Isaline Francey, Anna Surbone, Yannick Hurni

**Affiliations:** ^1^ Department of Gynecology and Obstetrics, Valais Hospital, Sion, Switzerland; ^2^ Department of Pediatrics, Gynecology and Obstetrics, Geneva University Hospitals, Geneva, Switzerland; ^3^ Department of General Surgery, Valais Hospital, Sion, Switzerland; ^4^ Department of Visceral Surgery, Geneva University Hospitals, Geneva, Switzerland; ^5^ Department of Oncology, Valais Hospital, Sion, Switzerland; ^6^ Department of Radio-Oncology, Valais Hospital, Sion, Switzerland; ^7^ Fertility Medicine and Gynaecologic Endocrinology Unit, Department Woman-Mother-Child, Lausanne University Hospital, Lausanne, Switzerland

**Keywords:** fertility preservation, ovarian transposition, pelvic radiotherapy, rectal cancer, uterine transposition, fertility sparing surgery

## Abstract

**Objective:**

To describe a case of utero-ovarian transposition (UOT) before pelvic radiation in a patient with rectal cancer and provide a systematic literature review on all reported cases of UOT.

**Methods:**

We performed a prospective collection and revision of clinical, intraoperative, and postoperative data from a patient who underwent UOT. In addition, a systematic review of the literature available to date on all cases of UOT was realized, and 14 patients from 10 articles were included.

**Results:**

We reported the case of a 28-year-old nulligravida patient who was diagnosed with a low-grade rectal adenocarcinoma and underwent neoadjuvant chemoradiotherapy, followed by transanal total mesorectal excision (TaTME). Before starting neoadjuvant oncological therapies, the patient underwent laparoscopic UOT. The intervention was performed without complications, and the patient received neoadjuvant oncological treatments as planned. TaTME and uterus repositioning were completed six weeks after the end of radiotherapy. No complications were observed during the first 9 postoperative months. Adequate utero-ovarian perfusion was assessed by Doppler ultrasound, cervicovaginal anastomosis appeared to have healed correctly, and the patient experienced menstrual bleeding. Data from the literature review of all reported cases of UOT were presented and discussed.

**Conclusions:**

UOT represents a valuable option to preserve fertility in patients requiring pelvic radiotherapy. This study provides additional evidence on the feasibility and safety of performing UOT.

## 1 Introduction

Colorectal cancer represents the third most common cancer and the second leading cause of cancer-related mortality worldwide (1). While colon and rectal cancer are often grouped, the incidence of rectal cancer is rising faster and is increasing among young adults (2). Due to advances in diagnosis and treatment, most young patients with rectal cancer present long-term survival, and many achieve an average life span (3). Long-term survivors are at risk of presenting chronic late effects resulting from cancer treatment, among which treatment-related infertility represents one of the principal but largely unaddressed problems (4). In addition, developed countries observe an increase in the average age of conception and delivery for women (5), raising the probability of patients being diagnosed with rectal cancer before completing family planning.

Fertility preservation is essential in managing young women requiring chemo- and radiotherapy for rectal cancer and other oncological diseases. Ovaries and oocytes are very sensitive to radiation and chemotherapeutic agents, and current fertility preservation strategies include oocytes, embryos, or ovarian tissue cryopreservation and ovarian transposition out of the radiation field (6). Nevertheless, patients have little probability of procreating due to irreversible uterine radiation damages, such as decreased volume and reduced distensibility due to myometrial fibrosis, vascular alterations, and endometrial injuries (7, 8). Pregnancy surrogacy was the only alternative until Ribeiro et al. first reported successful utero-ovarian transposition (UOT) in 2017 (9). This surgical technique protects the uterus by mobilizing it out of the radiation field, followed by reimplantation after radiotherapy. Since its first description, UOT has been reported less than 25 times, with only 2 cases performed in patients with rectal cancer (9–17). UOT remains an experimental approach, and all reported cases are essential to improve the knowledge concerning this procedure. In this study, we report successful UOT in a patient with rectal cancer.

## 2 Methods

**Figure 1:**
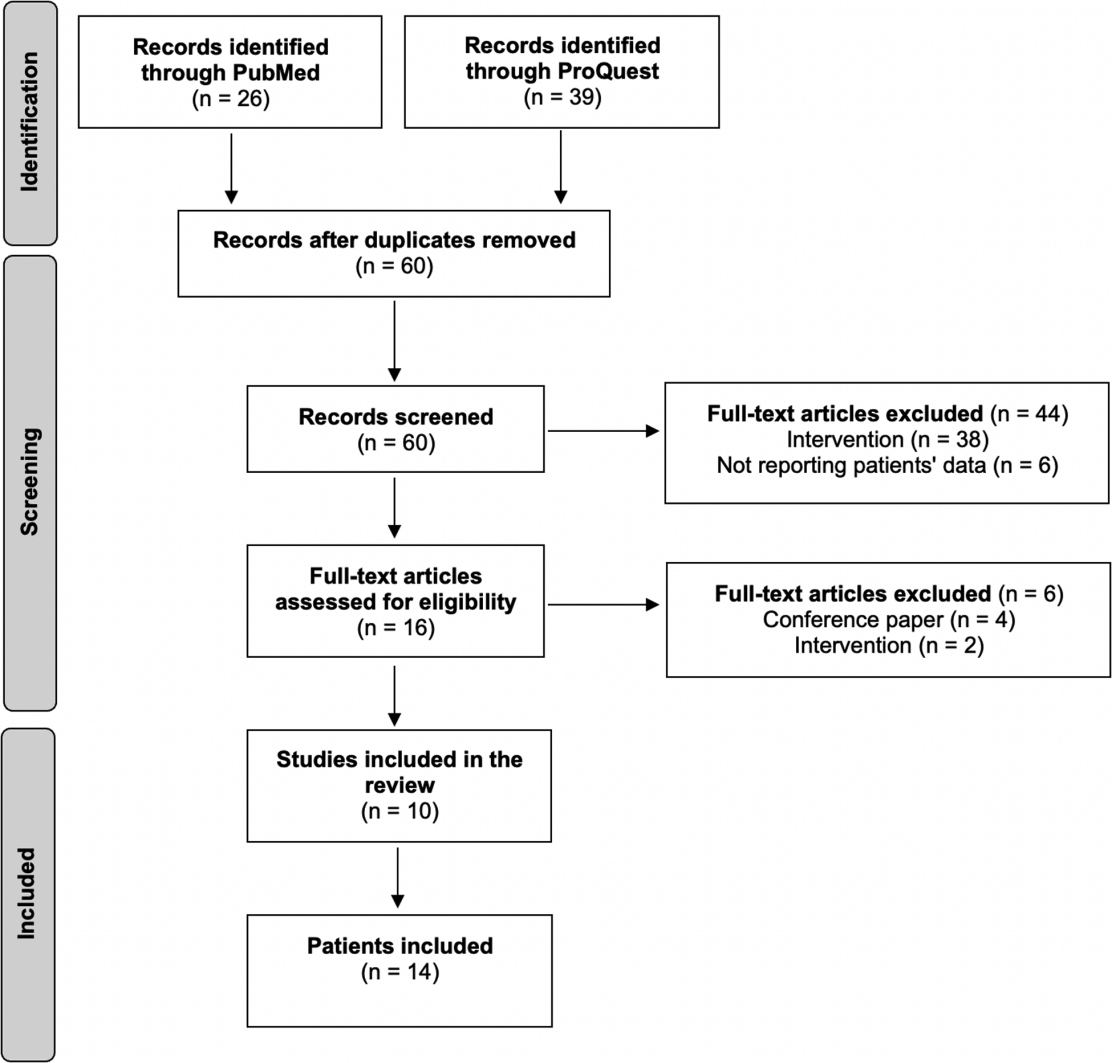
Selection flowchart showing the inclusion and exclusion process.

We prospectively collected and reviewed clinical, intraoperative, and postoperative data from a patient who underwent UOT. In addition, we realized a systematic review of the literature available to date, which results are presented in the discussion section. The systematic literature review was conducted using a structured search protocol based on the PRIMSA criteria. To find all cases of utero-ovarian transposition, PubMed and ProQuest databases were searched using the terms “uterine transposition”, “uterus transposition”, “uteroovarian transposition”, and “utero-ovarian transposition”. We included all articles in English, French, Italian, Spanish or Portuguese reporting at least 1 case of UOT. We excluded articles without individual data and articles with unavailable full text. We included 14 patients from 10 articles (9–18). The literature search protocol design is summarized in Figure 1.

## 3 Case report

### 3.1 Case presentation

A 28-year-old nulligravida patient presented with rectal bleeding and was diagnosed with a low-grade rectal adenocarcinoma located 7 cm from the anal margin. Magnetic resonance imaging and transrectal ultrasound showed a tumor of 4 cm in diameter infiltrating through muscularis propria into perirectal tissue, with 3 suspicious infracentimetric perirectal lymph nodes but no other disease foci (uT3N1cM0).

According to the PRODIGE-23 protocol, the suggested oncological treatment consisted of neoadjuvant chemotherapy with FOLFIRINOX (oxaliplatin 85 mg/m^2^, irinotecan 180 mg/m^2^, leucovorin 400 mg/m^2^, and fluorouracil 2,400 mg/m^2^ intravenously) every 14 days for 6 cycles, and neoadjuvant chemoradiotherapy (50.4 Gy during 5.5 weeks, with a reduction in fields after 45 Gy and 825 mg/m^2^ concurrent oral capecitabine twice daily for 5 days per week), followed by transanal total mesorectal excision (TaTME) and adjuvant chemotherapy with modified FOLFOX-6 (intravenous oxaliplatin 85 mg/m^2^ and leucovorin 400 mg/m^2^, followed by intravenous 400 mg/m^2^ fluorouracil bolus and then continuous infusion at a dose of 2,400 mg/m^2^ over 46 h every 14 days for six cycles) for 3 months.

Prior to neoadjuvant oncological treatment, the patient underwent ovarian stimulation following a random start antagonist protocol with cryopreservation of 29 mature oocytes for fertility preservation. In addition, after 4 cycles of FOLFIRINOX chemotherapy, we performed laparoscopic UOT to minimize utero-ovarian irradiation during radiotherapy (Supplementary Material Video S1).

### 3.2 Utero-ovarian transposition

**Figure 2:**
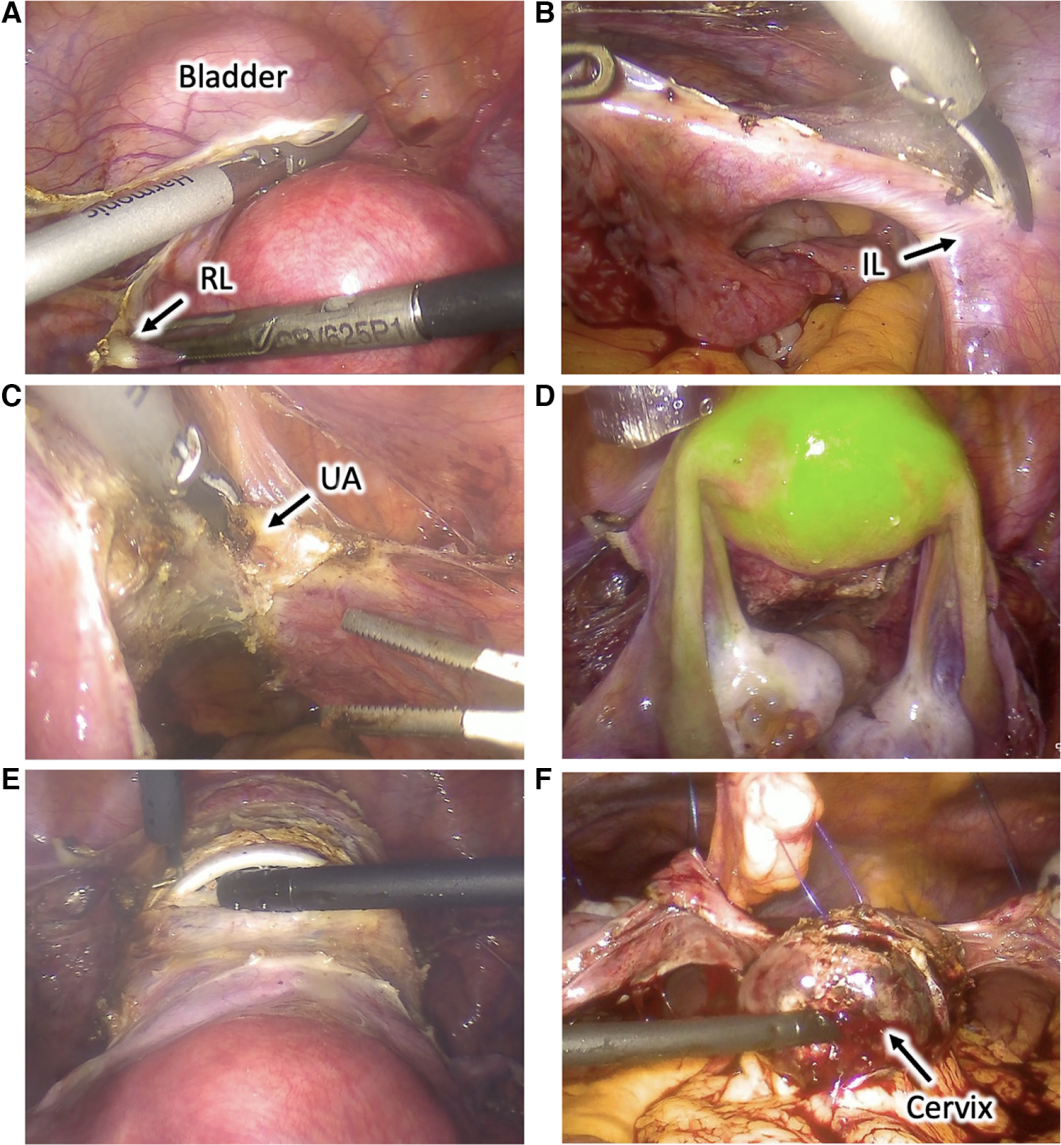
Utero-ovarian transposition. (**A**) The round ligaments are transected, the anterior leaves of the broad ligaments are dissected caudally, and the vesicouterine space is dissected to mobilize the bladder and expose the anterior vagina. (**B**) The infundibulopelvic ligaments are dissected and mobilized. (**C**) Uterine vessels are coagulated and cut at the uterine pedicles. (**D**) Uterus and adnexa perfusion is assessed using near-infrared fluorescence technology with an intravenous injection of indocyanine green. (**E**) Cardinal and uterosacral ligaments are sectioned, and a circular colpotomy is performed at the level of the cervicovaginal junction. (**F**) The uterus and adnexa are transposed to the upper abdomen and fixed to the anterior abdominal wall with transabdominal sutures. *RL, round ligament; IL, infundibulopelvic ligament; UA, uterine artery*.

The patient was placed in a dorsal lithotomy position under general anesthesia. A urinary catheter was placed, and a uterine manipulator was inserted. Access to the peritoneal cavity was achieved through a 12-mm umbilical trocar and two 5-mm right and left iliac trocars. The abdominal cavity inspection was unremarkable, and the patient was placed in a Trendelenburg position. To perform the surgery, we used conventional laparoscopic instruments with monopolar, bipolar, and ultrasonic energies (Figure 2).

The round ligaments were transected at the pelvic wall, the anterior leaves of the broad ligaments were dissected caudally to reach the vesicouterine fold, and their posterior leaves were sectioned up to the uterosacral ligaments. Vesicouterine space was dissected to mobilize the bladder and to expose the anterior vagina up to 1 cm distal to the cervicovaginal junction. Uterine vessels were coagulated and cut at the uterine pedicles. Cardinal and uterosacral ligaments were sectioned near the uterus, and a circular colpotomy was performed at the level of the cervicovaginal junction. The colpotomy was closed with a running suture using a Stratafix Spiral PDS 0. The uterus and the adnexa were completely mobilized into the pelvis. Their proper perfusion through the ovarian vessels was confirmed using near-infrared fluorescence technology with an intravenous injection of indocyanine green (ICG). The left and right colons were mobilized through the dissection along the Toldt's fascia to access the abdominal part of the ovarian vessels and allow their complete dissection and mobilization.

The uterus and adnexa were then transposed to the upper abdomen and fixed to the anterior abdominal wall with transabdominal sutures using PDS 0. Transabdominal sutures were fixed on the round and broad ligaments and the uterine isthmus. Periovarian tissue was marked with metallic clips allowing proper ovarian identification during radiotherapy. Proper utero-ovarian perfusion was confirmed again at the end of the procedure. The procedure lasted 3.5 h, and the estimated blood loss was 200 ml. We observed no postoperative complications, and utero-ovarian perfusion was assessed daily through Doppler ultrasound exams during the hospitalization. The patient received gonadotropin-releasing hormone agonists to induce amenorrhea, prevent intraabdominal menstrual bleeding, and induce ovarian suppression to protect ovarian function during chemotherapy. The patient was discharged 6 days after surgery. Utero-ovarian perfusion was assessed weekly through Doppler ultrasound exams. Utero-ovarian suspension sutures were cut 2 weeks after the intervention. No complications were observed during 6 postoperative weeks, and the patient was able to undergo 2 more cycles with FOLFIRINOX chemotherapy. Twelve weeks after the intervention, the patient started pelvic radiotherapy with concurrent oral capecitabine for 5 weeks.

### 3.3 Utero-ovarian reimplantation

**Figure 3:**
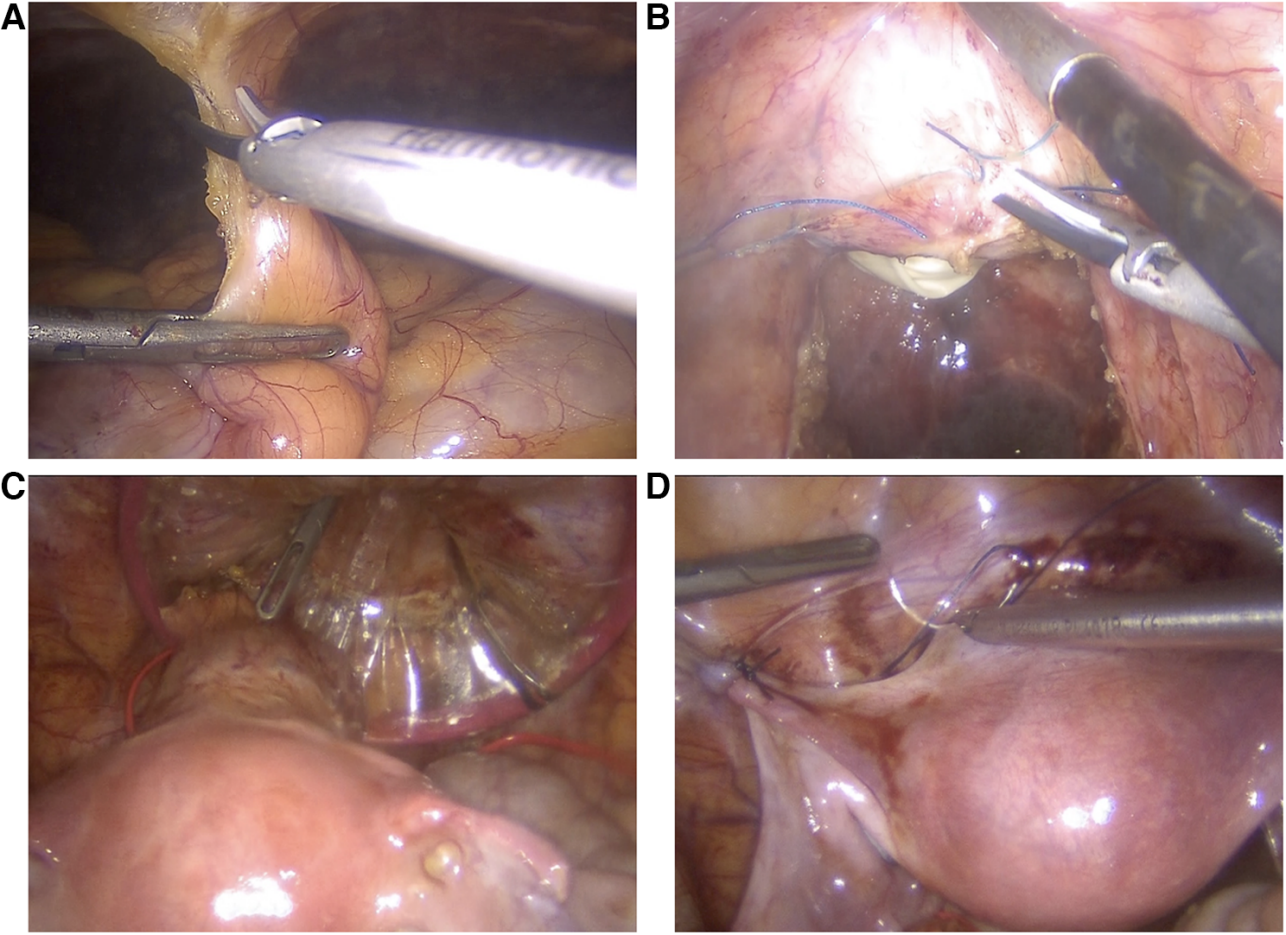
Utero-ovarian reimplantation. (**A**) The uterus and adnexa are freed from the adhesions, and attachments to the anterior abdominal wall are sectioned. (**B**) The vaginal vault is re-opened. (**C**) The uterus and adnexa are repositioned into the pelvis, and the cervix is introduced into the vagina through a Gelpoint vPath (9.5 cm) and sutured to it transvaginally. (**D**) The round and broad ligaments are sutured to the pelvic sidewall to their natural position.

Six weeks after the end of radiotherapy, TaTME and uterus repositioning were performed (Figure 3). Laparoscopic inspection showed normal-appearing uterus and adnexa. Their proper perfusion was confirmed using near-infrared fluorescence technology with an intravenous injection ICG. Adhesiolysis and sectioning of the uterine attachment to the anterior abdominal wall were performed.

TaTME was realized with end-to-end colorectal anastomosis and was associated with a discharge ileostomy. Vaginal vault opening was performed with a monopolar scalpel, and excised specimens were extracted transvaginally through a Gelpoint vPath (9.5 cm) (Applied Medical, Rancho Santa Margarita). The uterus and adnexa were repositioned into the pelvis, and the cervix was introduced into the vagina and sutured to it transvaginally with three contiguous running sutures using Vicryl 0. The round and broad ligaments were sutured to the pelvic sidewall to their natural position. The greater omentum was then mobilized, transposed into the pelvis, and interposed between the low rectal anastomosis and the cervicovaginal anastomosis to reduce the risk of fistulization. The procedure lasted 6 h, and the estimated blood loss was 100 ml. The ileostomy was closed nine days later, and the patient was discharged 4 days later.

### 3.4 Follow-up

No complications were observed during the first 9 postoperative months. Adequate utero-ovarian perfusion was assessed by Doppler ultrasound, and cervicovaginal anastomosis appeared healed correctly. Although the patient received oral contraception with a desogestrel-only pill, she presented irregular vaginal bleeding, testifying a preserved endometrial function. Definitive pathology showed a complete rectal tumor regression after neoadjuvant treatment (ypT0ypTN0), and in agreement with the patient, we decided not to administer adjuvant chemotherapy. Oncologic surveillance was planned, and the patient was advised to avoid getting pregnant during the first 12 postoperative months.

## 4 Discussion

We report a successful UOT in a patient with rectal cancer. Since 2017, this technique has been proposed as a fertility preservation method for selected patients requiring pelvic radiotherapy for colorectal (9, 14, 18), vaginal (12), and cervical cancers (10–12, 15, 17), intergluteal yolk sac tumors (13), and iliac myxoid low-grade liposarcoma (16). A total of 14 cases have been reported in 10 articles (Table 1). In addition, as reported in a congress paper, Ribeiro et al. performed UOT in 11 further patients with non-gynecological cancers (19). Since this represents the largest case series of UOT, we decided to report these data, but due to the limited information, they have not been integrated into the table.

**Table 1:** Clinicopathologic data on all reported cases of utero-ovarian transposition.

Case nr.	Reference	Age	Diagnosis (*TNM/FIGO staging*)	Oncological treatment	Utero-ovarian transposition	Utero-ovarian reimplantation	Follow-up (*months*)	Complications	Regular menses on follow-up	Oncological status
1	Ribeiro et al. (9)	26	Rectal adenocarcinoma (T3N1M0)	ChT, pelvic RT, and laparoscopic rectosigmoidectomy with total mesorectal excision	CL with CU-A	CL	18	Vaginal cuff dehiscence and migration of the left adnexa to the lower abdomen after UOT with ovarian exposure to radiotherapy	Yes	NED
2	Baiocchi et al. (10)	33	Squamous cervical cancer (T1b1N0M0/FIGO IB1)	Radical trachelectomy and pelvic RT	CL, no CU-A	CL	6	None	Yes	NED
3	Marques et al. (11)	28	Squamous cervical cancer (T1a1N1miM0/FIGO IIIC1p)	Conization, SLNB + pelvic LND, ChT and pelvic RT	RAL, no CU-A	RAL	20	After 12-months, cervical stenosis with fibrotic tissue resection and dilatation	Yes	NED
4	Baiocchi et al. (12)	32	Squamous cervical cancer (T1b2N0M0/FIGO IB2)	Radical trachelectomy, SLNB, pelvic RT	CL, no CU-A	CL	30	nd	Yes	NED
5	Baiocchi et al. (12)	29	Squamous cervical cancer (T1a2N1miM0/FIGO IIIC1p)	Radical trachelectomy, SLNB, pelvic RT	CL, no CU-A	The patient declined future fertility and underwent a simple hysterectomy	27	nd	NA	NED
6	Baiocchi et al. (12)	28	Squamous cervical cancer (T1a1N1miM0/FIGO IIIC1p)	Radical trachelectomy, SLNB + pelvic LND, pelvic RT	RAL, no CU-A	RAL	25	nd	Yes	NED
7	Baiocchi et al. (12)	38	Squamous cervical cancer (T1a2N1miM0/FIGO IIIC1p)	Radical trachelectomy, SLNB + pelvic LND, pelvic RT	CL, no CU-A	CL	1	nd	Yes	NED
8	Baiocchi et al. (12)	33	Squamous vaginal cancer (FIGO IIB)	Primary ChT and pelvic RT	CL, no CU-A	CL	2	nd	Yes	NED
9	Odetto et al. (17)	27	Squamous cervical cancer (T1b1N0M0/FIGO IB1)	Radical trachelectomy, SLNB, pelvic RT	CL, no CU-A	CL	12	None	Yes	NED
10	Vieira et al. (13)	3	Intergluteal yolk sac tumor	Tumor resection, systemic chemotherapy, and pelvic RT	CL, no CU-A	CL	15	None	No	NED
11	Kohler et al. (14)	40	Rectal adenocarcinoma (T3N1M0)	ChT, pelvic RT, and laparoscopic rectosigmoidectomy with total mesorectal excision	CL with CU-A	CL	nd	nd	nd	nd
12	Chernyshova et al. (15)	29	Squamous cervical cancer (T1b2N0M0/FIGO IB2)	Radical trachelectomy, SLNB + pelvic LND, pelvic RT, systemic ChT	LS, no CU-A	LS	25	None	Yes	NED
13	Ribeiro et al. (16)	28	Left iliac and thoracic synchronous myxoid low-grade liposarcoma	Tumor resection and RT	CL	CL	39	None	Yes, followed by spontaneous pregnancy with live birth at 36 weeks of gestation	NED
14	Lopez et al. (18)	32	Rectal adenocarcinoma (T3N1cM0)	ChT, pelvic RT, and laparoscopic rectosigmoidectomy with total mesorectal excision	CL with CU-A	CL	22	Superficial necrosis of the cervical mucosa observed through the CU-A after uterine transposition, followed by its treatment and proper healing	Yes, followed by spontaneous pregnancy with live birth at 36 weeks of gestation	NED
15	Our case	28	Rectal adenocarcinoma (T3N1M0)	ChT, pelvic RT, and laparoscopic rectosigmoidectomy with total mesorectal excision	CL, no CU-A	CL	4	None	Yes	NED

ChT, chemotherapy; RT, radiotherapy; CL, conventional laparoscopy; CU-A, cervical-umbilical anastomosis; NED, no evidence of disease; RAL, robotic-assisted laparoscopy; SLNB, sentinel lymph node biopsy; nd, no data; LND, lymph node dissection; LS, laparotomic surgery.

Before the advent of UOT, the main fertility preservation methods comprised oocytes, embryos, or ovarian tissue cryopreservation and ovarian transposition (20). None of these approaches preserve uterine function, and patients requiring pelvic radiotherapy generally must recur to surrogate pregnancy, which is expensive and not available in different countries. Conversely, UOT protects the uterus from radiation to allow it to sustain a pregnancy. An alternative is uterine ventral fixation, which is an easier way to mobilize the uterus from the radiation field (21, 22). This technique proposes to fix the uterus to the anterior abdominal wall to move it away from the radiation field for radiotherapy administered for anal or low rectal cancers. Despite allowing to reduce the dose on the uterus, ventral fixation seems not to spare it completely from radiation (21), especially in the case of higher radiotherapy targets such as parametria, upper vagina, pelvic lymph nodes, and high rectal cancers. Another alternative is uterus transplantation, which has been successfully performed more than 80 times with more than 40 live births from women presenting different types of absolute uterine factor infertility (23). However, uterus transplantation has never been performed after pelvic radiotherapy and is associated with significant disadvantages such as organ rejection, immunosuppressive therapy, surgical impact on living donors, the need for *in vitro* fertilization, and the required removal of the transplanted uterus after achieving the desired number of children or for complications (23, 24).

UOT is generally performed by conventional laparoscopy (9, 10, 12–14, 17), but endoscopic robotic surgery (11, 12) and laparotomic (15) procedures have also been employed. The technique involves mobilizing the uterus and adnexa from the pelvis, allowing their transposition to the upper abdomen to be fixed to the anterior abdominal wall. All utero-ovarian connections to the pelvis are sectioned except for the infundibulopelvic ligaments, which are released to allow proper UOT. Since uterine arteries are sectioned, utero-ovarian vascularization is only provided by the ovarian vessels. Perfusion can be evaluated intraoperatively using near-infrared fluorescence technologies with ICG (14) and postoperatively through Doppler ultrasound exams. The surgical technique to perform UOT appears relatively easy for most gyneco-oncological surgeons, who often dissect retroperitoneal structures such as the infundibulopelvic ligament. Some technical variations have been proposed starting from the original technique proposed by Ribeiro et al. (4). They originally proposed externalizing the cervix through the umbilicus to allow easy clinical evaluation of uterine perfusion and to permit menstrual bleeding exteriorization. Conversely, as in our case, the entire uterus and adnexa are more often let into the abdominal cavity (10–13, 15). In this case, surgery is more accessible and faster, and patients do not have to experience unpleasant umbilical bleeding and cervical secretions. GnRH agonists are generally administrated during chemotherapy to induce ovarian suppression and reduce the risk of gonadotoxicity (25). These also induce amenorrhea, avoiding intrabdominal menstruation. Suturing the ovaries to the posterior uterine wall, is another variation (26) proposed to reduce the risks of ovarian migration to the lower abdomen with consequent radiation exposure (9). UOT has also been successfully performed in a case of a 3-year-old patient, suggesting its feasibility in pre-pubertal patients (13).

Interventions for UOT and their reimplantation seem not to interfere with onco-surgical procedures. In our case, utero-ovarian reimplantation was performed at the same time that rectal resection without impeding its proper realization with complete mesorectal excision [as defined by Quirke (27)] and distant circumferential resection margins.

Cervical stenosis is a potential complication associated with UOT, especially in the case of trachelectomy for cervical cancer (11, 12, 17, 19). The partial dehiscence of uterine anastomosis needing re-suturing was reported once (12). Another potential complication could be the loss of uterine and ovarian reproductive functions or even their necrosis due to insufficient perfusion from the gonadal vessels. Although uterine viability with a preserved reproductive function has been proven following uterine arteries section for radical trachelectomies (28, 29), with postoperative uterine necrosis being observed in <1% of cases (30), the utero-ovarian function could be impaired by perfusion issues associated with their transposition to the upper abdomen. This is suggested by studies on patients who underwent ovarian transposition without concomitant radiotherapy, who present ovarian function disorders in around 10% of cases (31). Perfusion issues (e.g., thrombosis) associated with the dissection of infundibulopelvic ligaments, their mobilization, and the alteration of the anatomical path of their vessels could be responsible for these functional disorders. Uterine necrosis after UOT has been reported only once (19), but the risk of less serious postoperative utero-ovarian functional disorders could not be excluded, even if their potential incidence is currently difficult to predict. Low-molecular-weight-heparin, with or without aspirin, has been administered to mitigate the risk of thrombosis and subsequent utero-ovarian hypoperfusion (16, 26). Another potential risk is to move cancer cells from the pelvis to the upper abdomen. Due to the limited number of reported cases of UOT and their relatively short follow-up, this risk is currently difficult to evaluate. Currently, only one case of a patient's death from cancer progression with carcinomatosis for non-gynecological cancer 4 months after UOT was reported (19).

Recently, Ribeiro et al. reported the first case of live birth after UOT (16). This patient, diagnosed with liposarcoma, got spontaneously pregnant 2 years after UOT and had an uneventful pregnancy until 36 weeks of gestation, when she presented preterm labor and underwent a cesarean section, with good maternal and neonatal outcomes (16). Afterward, Lopez et al. reported the case of another live birth following a spontaneous pregnancy after UOT in a patient diagnosed with rectal carcinoma (18). In addition, a third case of live birth after UOT performed by the same surgical team in a patient with cervical cancer was reported through the mass media.

This is a proof-of-concept for the feasibility of UOT to prevent infertility in patients requiring pelvic radiotherapy. Despite this encouraging result, UOT remains an experimental approach, and more studies are needed before proposing this approach to a larger number of patients. UOT should be proposed only in selected cases, and patients should be aware of some unresolved issues, such as long-term oncological safety and the effective ability to procreate after this intervention. In addition to UOT, patients must be offered the standard fertility preservation methods, such as oocytes/embryos cryopreservation, to allow for *in vitro* fertilization in the case of a lack of spontaneous conceptions or pregnancy surrogacy for failed uterine preservation.

In conclusion, UOT represents a valuable option to preserve fertility in patients requiring pelvic radiotherapy. However, knowledge is still limited, and this study provides a summary of the reported cases so far, in addition to further evidence on the feasibility and safety of performing UOT.

## References

[1] Bray F Ferlay J Soerjomataram I Siegel RL Torre LA Jemal A. Global cancer statistics 2018: globocan estimates of incidence and mortality worldwide for 36 cancers in 185 countries. CA Cancer J Clin. (2018) 68(6):394–424. 10.3322/caac.21492 30207593

[2] Siegel RL Fedewa SA Anderson WF Miller KD Ma J Rosenberg PS Colorectal cancer incidence patterns in the United States, 1974–2013. J Natl Cancer Inst. (2017) 109(8):djw322. 10.1093/jnci/djw322 PMC6059239 28376186

[3] Siegel RL Miller KD Goding Sauer A Fedewa SA Butterly LF Anderson JC Colorectal cancer statistics, 2020. CA Cancer J Clin. (2020) 70(3):145–64. 10.3322/caac.21601 32133645

[4] Stal J Yi SY Cohen-Cutler S Gallagher P Barzi A Freyer DR Fertility preservation discussions between young adult rectal cancer survivors and their providers: sex-specific prevalence and correlates. Oncologist. (2022) 27(7):579–86. 10.1093/oncolo/oyac052 35427410 PMC9255970

[5] United nations population division d of e and sa. World fertility patterns 2015–data booklet (st/esa/ser. A/370) (2015).

[6] Christianson MS Oktay K. Advances in fertility-preservation surgery: navigating new frontiers. Fertil Steril. (2019) 112(3):438–45. 10.1016/j.fertnstert.2019.06.029 31446903

[7] Wo JY Viswanathan AN. Impact of radiotherapy on fertility, pregnancy, and neonatal outcomes in female cancer patients. Int J Radiat Oncol Biol Phys. (2009) 73(5):1304–12. 10.1016/j.ijrobp.2008.12.016 19306747 PMC2865903

[8] Teh WT Stern C Chander S Hickey M. The impact of uterine radiation on subsequent fertility and pregnancy outcomes. Biomed Res Int. (2014) 2014:482968. 10.1155/2014/482968 25165706 PMC4140124

[9] Ribeiro R Rebolho JC Tsumanuma FK Brandalize GG Trippia CH Saab KA. Uterine transposition: technique and a case report. Fertil Steril. (2017) 108(2):320–4.e1. 10.1016/j.fertnstert.2017.06.016 28697913

[10] Baiocchi G Mantoan H Chen MJ Faloppa CC. Uterine transposition after radical trachelectomy. Gynecol Oncol. (2018) 150(2):387–8. 10.1016/j.ygyno.2018.05.009 29803317

[11] Marques RM Tsunoda AT Dias RS Pimenta JM Linhares JC Ribeiro R. Robotic uterine transposition for a cervical cancer patient with pelvic micrometastases after conization and pelvic lymphadenectomy. Int J Gynecol Cancer. (2020) 30(6):898–9. 10.1136/ijgc-2020-001250 32430386

[12] Baiocchi G Vieira M Moretti-Marques R Mantoan H Faloppa C Damasceno RCF Uterine transposition for gynecological cancers. Int J Gynecol Cancer. (2021) 31(3):442–6. 10.1136/ijgc-2020-001780 33649011

[13] Vieira MA Vieira AGS Fonseca DSL Jorge GE Lopes LF Ribeiro RC. Uterine transposition in a pre-pubertal patient. Int J Gynecol Cancer. (2021) 31(3):492–3. 10.1136/ijgc-2020-002074 33649020

[14] Kohler C Kettner P Arnold D Puhl G Marnitz S Plaikner A. Repeated intravenous indocyanine green application to prove uterine perfusion during uterus transposition. Int J Gynecol Cancer. (2022) 32(11):1479–80. 10.1136/ijgc-2022-003647 35764348

[15] Chernyshova A Marchenko E Chekalkin T Kolomiets L Chernov V. Performing a radical trachelectomy with uterine transposition in a patient with stage ib2 cervical cancer: a case report. Cancer Treat Res Commun. (2023) 34:100681. 10.1016/j.ctarc.2023.100681 36638644

[16] Ribeiro R Anselmi MC Schneider GA Furtado JPR Shwareb MGMA Linhares JC. First live birth after uterine transposition. Fertil Steril. (2023) 120(1):188–93. 10.1016/j.fertnstert.2023.02.033 36863432

[17] Odetto D Saadi JM Chacon CB Wernicke A Ribeiro R. Uterine transposition after radical trachelectomy. Int J Gynecol Cancer. (2021) 31(10):1374–9. 10.1136/ijgc-2021-002944 34607821

[18] Lopez A Perez Villena JF Guevara Jabiles A Davila K Sernaque Quintana R Ribeiro R. Uterine transposition and successful pregnancy in a patient with rectal cancer. Int J Gynecol Cancer. (2023) 33(8):1310–5. 10.1136/ijgc-2023-004661 37549972

[19] Ribeiro R Baiocchi G Moretti-Marques R Tsunoda A Linhares J Pareja R. O002/#185 uterine transposition: feasibility study. Int J Gynecol Cancer. (2022) 32(Suppl 3):A3. 10.1136/ijgc-2022-igcs.4

[20] Oktay K Harvey BE Partridge AH Quinn GP Reinecke J Taylor HS Fertility preservation in patients with cancer: asco clinical practice guideline update. J Clin Oncol. (2018) 36(19):1994–2001. 10.1200/JCO.2018.78.1914 29620997

[21] Köhler C Marnitz S Biel P Cordes T. Successful delivery in a 39-year-old patient with anal cancer after fertility-preserving surgery followed by primary chemoradiation and low anti-mullerian hormone level. Oncology. (2016) 91(5):295–8. 10.1159/000449416 27677176

[22] Azaïs H Canova CH Vesale E Simon JM Canlorbe G Uzan C. Laparoscopic uterine fixation to spare fertility before pelvic radiation therapy. Fertil Steril. (2018) 110(5):974–5. 10.1016/j.fertnstert.2018.07.020 30316445

[23] Brännström M Racowsky C Carbonnel M Wu J Gargiulo A Adashi EY Uterus transplantation: from research, through human trials and into the future. Hum Reprod Update. (2023) 29:109–16. 10.1093/humupd/dmad012 PMC10477946 37328434

[24] Brännström M Dahm-Kähler P. Uterus transplantation and fertility preservation. Best Pract Res Clin Obstet Gynaecol. (2019) 55:109–16. 10.1016/j.bpobgyn.2018.12.006 30711374

[25] Arecco L Ruelle T Martelli V Boutros A Latocca MM Spinaci S How to protect ovarian function before and during chemotherapy? J Clin Med. (2021) 10(18):4192. 10.3390/jcm10184192 34575299 PMC8467797

[26] Ribeiro R Baiocchi G Tsunoda AT Linhares JC Pareja R. Uterine transposition technique: update and review. Minerva Ginecol. (2019) 71(1):62–71. 10.23736/S0026-4784.18.04360-5 30486638

[27] Quirke P Durdey P Dixon MF Williams NS. Local recurrence of rectal adenocarcinoma due to inadequate surgical resection. Histopathological study of lateral tumour spread and surgical excision. Lancet. (1986) 2(8514):996–9. 10.1016/S0140-6736(86)92612-7 2430152

[28] Escobar PF Ramirez PT Garcia Ocasio RE Pareja R Zimberg S Sprague M Utility of indocyanine green (icg) intra-operative angiography to determine uterine vascular perfusion at the time of radical trachelectomy. Gynecol Oncol. (2016) 143(2):357–61. 10.1016/j.ygyno.2016.08.239 27544455 PMC5966828

[29] Tang J Li J Wang S Zhang D Wu X. On what scale does it benefit the patients if uterine arteries were preserved during art? Gynecol Oncol. (2014) 134(1):154–9. 10.1016/j.ygyno.2014.04.043 24786639

[30] Vieira MA Rendón GJ Munsell M Echeverri L Frumovitz M Schmeler KM Radical trachelectomy in early-stage cervical cancer: a comparison of laparotomy and minimally invasive surgery. Gynecol Oncol. (2015) 138(3):585–9. 10.1016/j.ygyno.2015.06.023 26095894

[31] Laios A Otify M Papadopoulou A Gallos ID Ind T. Outcomes of ovarian transposition in cervical cancer; an updated meta-analysis. BMC Womens Health. (2022) 22(1):305. 10.1186/s12905-022-01887-8 35869476 PMC9308360

