# Supplementary figures and images for: Utero-ovarian transposition before pelvic radiation in a patient with rectal cancer: a case report and systemic literature review
Source: Front Surg. 2024 Feb 26;11:1336047. doi: 10.3389/fsurg.2024.1336047 (PMC10925680; doi:10.3389/fsurg.2024.1336047)

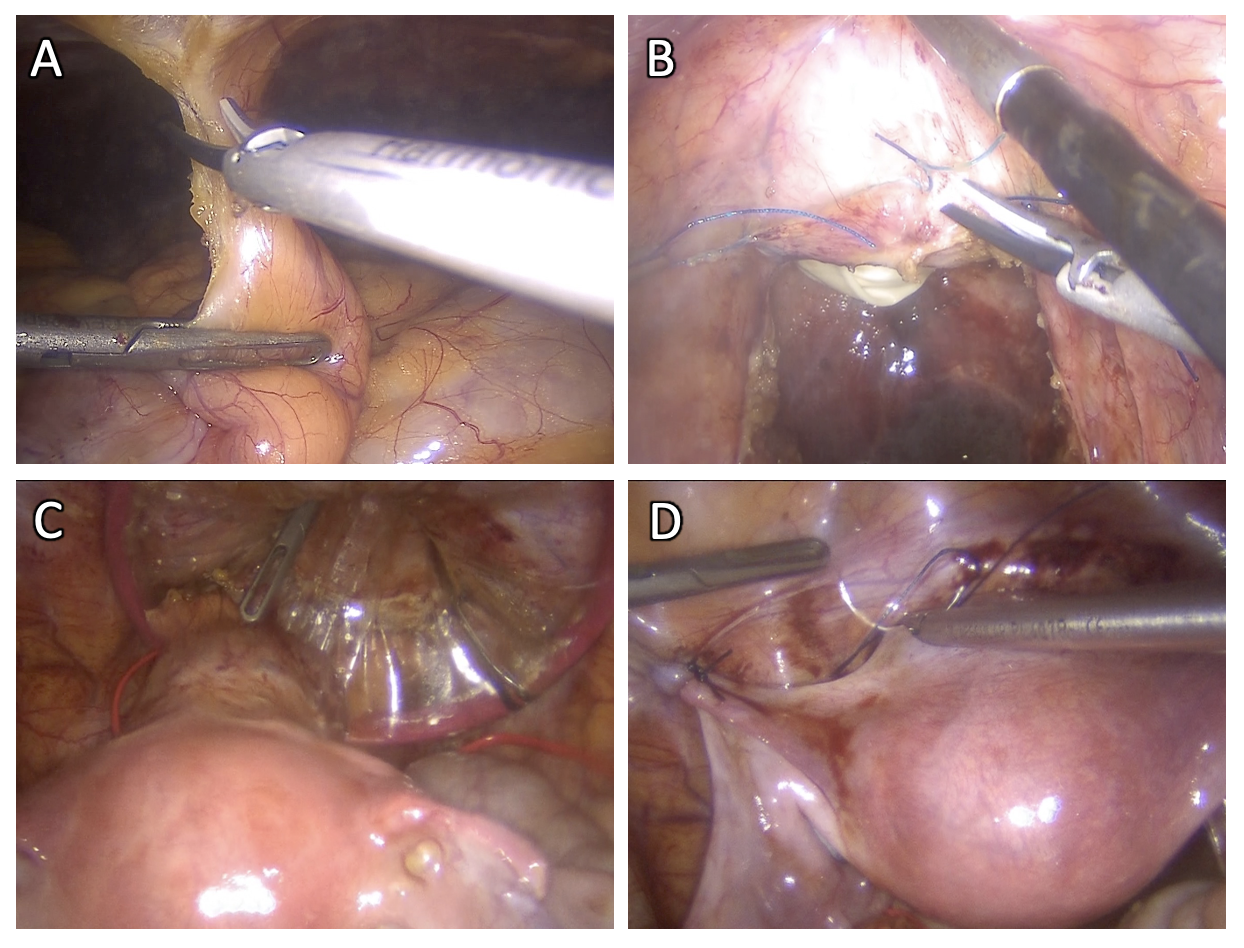

Supplement: Supplementary file 2 [file Image1.tiff]

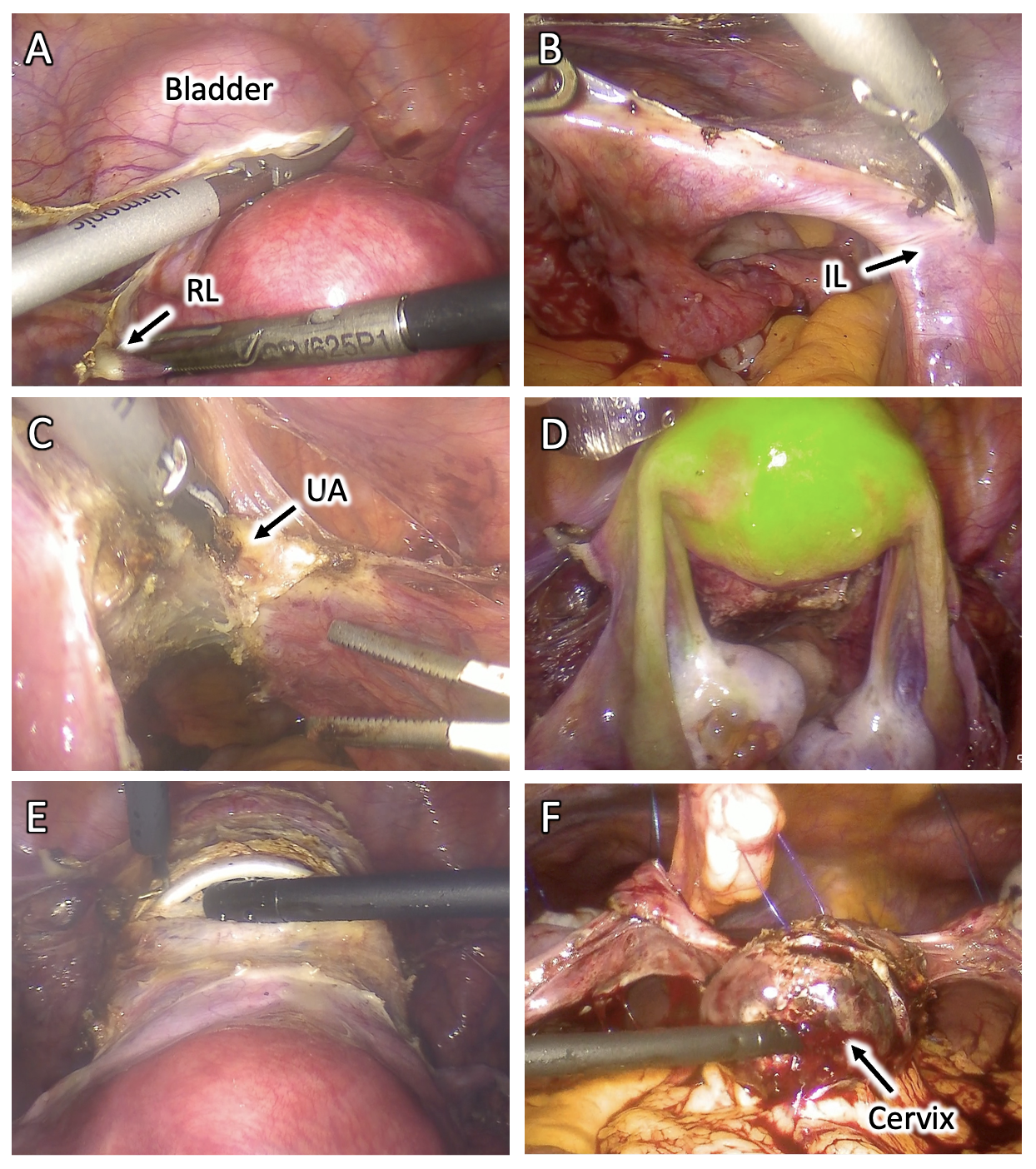

Supplement: Supplementary file 3 [file Image2.tiff]
